# Assessment of depression in veterans across missions: a validity study using Rasch measurement models

**DOI:** 10.1080/20008198.2017.1326798

**Published:** 2017-05-22

**Authors:** Karen-Inge Karstoft, Anni B. S. Nielsen, Tine Nielsen

**Affiliations:** ^a^ Research and Knowledge Centre, The Danish Veteran Centre, Ringsted, Denmark; ^b^ The Research Unit and Section of General Practice, Institute of Public Health, University of Copenhagen, Copenhagen, Denmark; ^c^ Department of Psychology, University of Copenhagen, Copenhagen, Denmark

**Keywords:** Item response theory, Rasch model, depression, military personnel, psychometrics, rash decisions

## Abstract

**Background**: Depression is a common psychopathological outcome following military deployment. Previous studies have reported differing rates of post-deployment depression, indicating that the toll of war differs across missions. However, it is unclear to what degree the varying prevalence is due methodological differences. Studies comparing rates of depression across cohorts using the same methodology and ensuring measurement invariance are rare, leaving us with limited knowledge on the actual depression prevalence variance across missions.

**Objective**: Applying Rasch models (RM), we aim to validate a measure of depression distributed to all personnel deployed with the Danish Defense since 1998. The main focus was establishing a sufficient sum score and measurement invariance relative to deployment cohort.

**Method**: Two cohorts of the International Security Assistance Force (ISAF) deployed to Afghanistan in 2009 (ISAF7, *N* = 265) and 2013 (ISAF15, *N* = 271) were included. Participants filled out a questionnaire concerning their Psychological Reactions to International Missions (PRIM) approximately seven months after home-coming. The questionnaire included a 10-item scale of depression symptoms (PRIM-Depression). The validity of the PRIM-Depression was tested using RM with specific focus on differential item functioning (DIF) across the two cohorts.

**Results**: The PRIM-Depression scale displayed excellent overall consistency and showed no problems with monotonicity or homogeneity. However, the full PRIM-Depression scale did not fit a pure RM. We therefore tested the fit of items to a graphical log-linear RM and found evidence of DIF for two items relative to cohort. We proceeded without these two items and tested the resulting 8-item version which fitted a pure RM without DIF on any of the exogenous variables.

**Conclusions**: Our results suggest that the 10-item PRIM-Depression scale should be used to compare cohorts only with appropriate score equation. The 8-item version provides a sufficient statistic and can as such be applied using the raw score.

## 1. Introduction

Substantial proportions of soldiers returning from deployment in war zones go on to develop mental health problems (2–17% across studies; Richardson, Frueh, & Acierno, 2010). Posttraumatic stress disorder (PTSD) is one of the most investigated mental health consequences of military deployment. However, approximately 40% of those evaluated after deployment are not diagnosed with PTSD but with other psychiatric disorders (Packnett, Gubata, Cowan, & Niebuhr, 2012; Piccirillo, Packnett, Boivin, & Cowan, 2015), most frequently depression or symptoms thereof (Stander, Thomsen, & Highfill-McRoy, 2014).

While depression after deployment often occurs comorbidly with PTSD (Stander et al., 2014; Van Hooff et al., 2014), it is also seen with no concurrent PTSD (O’Donnell, Creamer, & Pattison, 2004). Research results suggest post-deployment prevalence of depression with or without comorbid PTSD ranging from 2–15% (Hoge et al., 2004; Reijnen, Rademaker, Vermetten, & Geuze, 2015). This variance in prevalence across studies is hypothesized to partly reflect differences in sampling, assessment tools, time of assessment, attrition rates or other methodological issues such as unrecognized non-invariance of measurement (Ramchand et al., 2010). However, depression prevalence differences may also reflect an actual difference due to different combat exposure or specific mission characteristics. Disentangling methodological from actual differences is crucial in order to understand the toll on mental health that different wars and different missions cause among soldiers. However, studies that systematically compare rates of mental health problems across multiple cohorts using the same methodology while ensuring measurement invariance across these cohorts are rare (Richardson et al., 2010), leaving us with limited knowledge on how different deployments affect soldiers differently.

Since 1992, approximately 31,000 soldiers have been deployed to international missions with the Danish Military (Statistics from the Danish Veteran Centre’s deployment database). From 1998 onwards, the Psychological Reactions following deployment to International Missions (PRIM) questionnaire on post-deployment mental health has been distributed to all deployed soldiers 7–8 months after home-coming (Andersen, 1998). This large amount of systematically collected data provides a unique opportunity to compare the psychological toll of deployment across different cohorts, wars and missions with different levels and types of combat exposure.

Included in the PRIM is a subscale consisting of 10 items aimed at measuring symptoms of depression (PRIM-Depression, see Table 1). The scale was constructed by Danish military psychologists based on other depression questionnaires and the literature on depression (Andersen, 1998). However, no formal validation of the scale has been conducted and, hence, the reliability and validity of the scale can be questioned. Valid assessment tools for deployment- and psychotrauma-related outcomes are crucial to obtain valid results (Frewen, Dean, & Lanius, 2012; Olff, 2015).

**Table 1.** Items in the 10-item PRIM-Depression scale.*

*How often did you experience the following during the last three months?*
1. Were you easily saddened?
2. Did you have thoughts about taking your own life?
3. Did you have worries about the future?
4. Did you feel sad?
5. Did you feel inferior or insecure?
6. Did you feel empty inside?
7. Did you feel abandoned?
8. Did you worry a lot?
9. Did you feel like something inside was broken?
10. Did you feel that everything was meaningless?

*Items appear in Danish in the original questionnaire and have been translated for the purpose of this study.

Validation of psychological measurement scales and instruments have historically been, and are still, mainly conducted within the classical test theory (CTT) framework. However, in recent decades, there has been a growing number of studies employing modern test theory, particularly confirmatory factor analysis (CFA) focusing on the dimensionality issue of such instruments and, to a lesser degree, item-response models (IRT) focusing on the issue of item fit (Bentley, Gallagher, Carl, & Barlow, 2014; Tsai et al., 2015). IRT provides a solid foundation for assessing the accuracy and invariance of a scale in measuring the trait it intends to measure (Van der Linden & Hambleton, 2013). A special instantiation of IRT is the family of Rasch models (RM; Fischer & Molenaar, 2012), with the RM for dichotomous items, also known as the one-parameter or 1PL model, being the simplest (Rasch, 1960). The fit of a set of item responses to the RM provides ideal measurement in the specific frame of reference that the analysis is undertaken in (e.g. population or purpose), i.e. the score is a sufficient statistic which contains all the information on responses (Kreiner, 2013, 2007). This is only true for the RM, which is why it is particularly suited in settings where the raw score is being used. Accordingly, if the assumptions of a RM are met, including the requirement of invariance, the raw score of a scale is said to accurately reflect the degree of the trait it intends to measure (Da Rocha, Chachamovich, de Almeida Fleck, & Tennant, 2013; Hamon & Mesbah, 2002; Kreiner, 2013, 2007; Tennant & Conaghan, 2007). In the case of PRIM-Depression, a fit to a RM would imply that the raw PRIM-Depression scale score contains all the information required to assess the soldiers’ level of depression, and that no additional information such as age, gender or deployment history need be taken into consideration.

A validation of the PRIM-Depression Scale would provide clinicians in the Danish Military with a valuable tool for screening that can be easily used across cohorts, deployments and wars. A valid DPRIM-Depression instrument would enable accurate screening of soldiers after return from deployment, thus leading to early identification of those in need of treatment and allocation of treatment resources to those in need. In addition, it would provide a valuable data source for studying the level of depression among soldiers deployed to different war zones and different missions with differing levels of combat exposure.

In this study, we therefore aim to validate the PRIM-Depression measure in Danish military personnel after deployment to Afghanistan with the Danish Military using RM, with a particular emphasis on the issue of measurement invariance and the establishment of a sufficient sum score.

## 2. Methods

### 2.1. Participants

We include military personnel from two different cohorts deployed to Afghanistan as part of the International Security Assistance Force (ISAF), the first cohort in 2009 (Cohort 1) and the second one in 2013 (Cohort 2). Of all available cohorts, these two were of special interest because, in addition to the PRIM, they also received a series of validated questionnaires before and during deployment. Furthermore, while they deployed to the same war (Afghanistan) the threat assessment was expectedly different for the two cohorts, making them ideal for testing potential measurement bias due to differences in threat. We therefore selected these two cohorts for the current study. From the two we included everyone who had complete data for the 10 depression items, as well as for five relevant exogenous variables: gender, age, type of contract, previous deployments, and danger or injury during deployment. The total *N* for the two cohort samples used in the study is 536 (Cohort 1 = 265; Cohort 2 = 271). For Cohort 1, 95.1% were male and the mean age was 27.8 (*SD* = 7.7). For Cohort 2, 91.9% were male and the mean age was 31.5 (8.1). Cohort 1 had a of 20.4 (*SD* = 4.8) while Cohort 2 had a mean danger injury score of 15.9 (*SD* = 4.3). The differences between cohorts in age and danger injury score were significant (*p*s <.001) while the gender distribution was not significantly different between cohorts.

### 2.2. Instruments

#### 2.2.1. Depression items

The PRIM-Depression scale consists of 10 items (see Table 1) concerning symptoms of depression (sample item: I have been feeling sad). Items included in PRIM-Depression have high semantic overlap with items included in one or more known depression scales, for example ‘feeling sad’, ‘feeling inferior’ etc. (for comparison of all items see Fried, 2017). There are four response categories for each item: 0 = ‘no’ or ‘never’; 1 = ‘occasionally’; 2 = ‘quite often’; 3 = ‘very often’. For this study, item responses were dichotomized into 0 (symptom absent, previous category 0) and 1 (symptom present, covering the categories 1–3), as the PRIM-Depression scale is primarily used as a screening measure, and therefore it is important to identify everyone who endorses a symptom at any level versus those who do not.

#### 2.2.2. Exogenous variables

In order to evaluate measurement invariance (i.e. no differential item functioning [DIF]) we included a range of exogenous variables of importance for the depression assessment in formerly deployed military personnel: *Age* and *gender* have with some consistency been found to be related to psychopathology following deployment (Brewin, Andrews, & Valentine, 2000; Xue et al., 2015), and *previous deployments* have been found to increase the risk of depression following current deployment (Kline et al., 2010). These variables are therefore included for DIF analysis. *Type of contract* (permanent or temporary) has been less frequently investigated as a predictor of post-deployment mental health. However, in civilian populations, low perceived job security, e.g. having a temporary versus permanent contract, is associated with poorer mental health including depressive symptoms (Burr, Rauch, Rose, Tisch, & Tophoven, 2015; Virtanen, Vahtera, Kivimäki, Pentti, & Ferrie, 2002). Therefore, it seems reasonable to assume that differences in military experience for these two groups as well as differences in the mind-sets related to being or not being a career soldier potentially is associated with differences in post-deployment mental health. Combat exposure or exposure to other potentially traumatic event is according to diagnostic criteria central and necessary for the development of PTSD (American Psychiatric Association, 2000), and relevant for the development of related post-deployment mental health adversities. To capture this, we included a measure of perceived danger and injury during the deployment (Danger-Injury Scale; Berntsen et al., 2012). For the purpose of this study, we dichotomized the Danger-Injury score to indicate low/high exposure to danger (median used for cut point). Finally and most importantly, we included the deployment cohort as an exogenous variable so that any DIF dependent on cohort could be identified and adjusted for in future comparisons of depression levels across cohorts.

For comparison with existing depression measures, we used the available measures in the two cohorts, namely the Beck Depression Inventory (BDI; Beck, Steer, & Carbin, 1988) for Cohort 1 and the depression subscale of the Depression, Anxiety, and Stress Scales (DASS; Lovibond & Lovibond, 1995) for Cohort 2.

## 3. Analysis

### 3.1. Rasch measurement models

The RM for dichotomous items (Rasch, 1960) has five basic requirements for measurement (Kreiner, 2013): *Unidimensionality* (i.e. that the items of a scale measure only one underlying latent construct, in this case depression), *Monotonicity* (i.e. that the probability of a high item score increases with increasing values of the latent variable, in this case, higher scores on the PRIM-Depression items follow from higher levels of depression), *Homogeneity* (i.e. that the rank order of the item parameters (item ‘difficulties’) is the same for all persons regardless of their level on the latent variable, here level of depression), *no local dependence* (LD; i.e. that the items in a scale must be conditionally independent given the latent variable, in this case meaning that item scores are only dependent on the level of depression and not on affirmation of other items), and *absence of differential item functioning* (no DIF; i.e. that the items in a scale must be conditionally independent of exogenous variables given the latent variable, in this case, item level is dependent only on level of depression and not on exogenous variables such as age, gender, cohort, etc.).

Close to optimal measurement can still be achieved in cases where fit to the pure RM is rejected, provided that the departures from the RM consist only of uniform differential item functioning (uniform DIF) and/or uniform local dependency between items (uniform LD) (Kreiner & Christensen, 2007). Uniform DIF is present when item responses depend not only on the latent variable, but also on membership of a sub-group, that is when a sub-group is more inclined to endorse a particular item than are other subgroups independent of their level on the latent variable, and in the same way (i.e. uniform) across the range of the latent variable. Such departures of the model can be incorporated and adjusted for in a so-called graphical log-linear Rasch model (GLLRM), which is an extension of the RM that allows the specific departures of uniform LD and DIF. If the GLLRM incorporates DIF, the score can no longer be regarded as a sufficient statistic, as additional information on subgroup membership is needed to assess the level of a person on the latent variable correctly. When DIF is present, scores must be equated across the sub-groups to allow comparative analyses between subgroups (Kreiner, 2007).

### 3.2. Item analysis by RM and GLLRM

All item analyses were conducted with the same general strategy: the fit of the item responses to the pure RM was tested first and, if fit to the RM could not be established, we proceeded by testing whether item responses fitted a GLLRM with uniform LD and/or uniform DIF. Overall model fit (i.e. homogeneity of the item parameters in approximately equal sized score groups) was tested using Andersen’s (1973) Conditional Likelihood Ratio test (CLR), as was global DIF. Fit of individual items was tested by comparing observed item-test score correlations with expected item-test score correlations under the model (Kreiner, 2011).

In GLLRMs, the presence of LD and DIF was tested by conditional tests of independence by using partial Goodman-Kruskal gamma coefficients to measure the conditional association between item pairs (LD) or between items and exogenous variables (DIF) given the rest-scores (Kreiner & Christensen, 2004). Specifically, the presence of DIF was tested relative to gender and age group (the latter dichotomized, <30 years or ≥30 years), previous deployments (no, yes), type of contract (permanent, temporary), the level of danger they had been exposed to (low, high), and their deployment cohort (Cohort 1, Cohort 2).

Reliability was estimated using Cronbach’s alpha, when fit to an RM was established. When fit to a GLLRM was established, reliability was instead estimated using Hamon and Mesbah’s (2002) estimation method of reliability, which takes into account the departures from the RM. Targeting (Target is the value of the theta or observed scale where the test information is highest) was evaluated by two indices (Kreiner & Christensen, 2013): the test information target index (the mean test information divided by the maximum test information for theta) and the root mean squared error (RMSE) target index (the minimum standard error of measurement divided by mean standard error of measurement for theta). Both should preferably be close to a value of 1. The target of the observed score was estimated, as was the standard error of measurement of the observed score (SEM).

The original PRIM-Depression scale with ordinal items has, as mentioned above, been used as a screening instrument. The cutoff score of 16 on the non-dichotomized categories of the 10 items differentiates between non-cases and possible-cases to be referred for further assessment by military psychologists. Transforming the original cutoff score to the dichotomous depression scale gives a cutoff score of 4.66. Hence, we evaluated a cutoff of five, with individuals scoring 0–5 tentatively in the non-depression group, and individuals scoring 6–10 in the depression group. One way to evaluate the relevance of this cut point was to test whether, in the GLLRM for the full 10-item PRIM-Depression scale, the item parameters for the non-cases (below the cut point) and the possible-cases (above the cut point) were indeed different and, if so, to proceed with Rasch/graphical log-linear Rasch analyses of each of these groups separately to ascertain the psychometric properties of the scale for these two restricted score ranges, in the usual manner.

To enable comparison with existing measures of depression, we present correlation analyses of the best fitting scale and Beck Depression Inventory (BDI; Beck et al., 1988, for Cohort 1) and the depression subscale of the Depression, Anxiety and Stress Scale (DASS; Lovibond & Lovibond, 1995, for Cohort 2)

A critical level of *p* < .05 was used to imply statistical significance for all tests. However, as recommended by Cox et al. (1977), we did not use this critical value as a deterministic decision criterion. The Benjamini-Hochberg procedure was applied to correct for the false discovery rate (FDR) due to multiple testing, when appropriate (Benjamini & Hochberg, 1995).

The statistical software DIGRAM 3.04 (Kreiner & Nielsen, 2013) was used for all isis, as the implementation of GLLRM in this package provides formal tests for sufficiency, as well as analysis of DIF and LD, while adjusting for the false discovery rate due to multiple testing.

## 4. Results

### 4.1. Item analysis of the full 10-item PRIM-Depression scale

The raw distribution of PRIM-Depression scores across the two cohorts ranged from 0 to 10 with a mean of 3.27 (*SD* = 2.85) symptoms being endorsed by the deployed personnel (data not shown). The initial descriptive item analysis showed no problems with monotonicity or homogeneity of the 10 depression items. The consistency of the 10 depression items was excellent, with all item correlations (rank correlations as the items are ordinal) between 0.68 and 0.96, and all item-rest score correlations between 0.71 and 0.87.

The 10-item PRIM-Depression scale did not fit the pure RM (Table 2). Further analysis showed the PRIM-Depression scale to fit a GLLRM (Table 2), with evidence only for DIF for item 3 and item 8 relative to cohort (*p *< .001 in both cases). No evidence of additional DIF relative to degree of danger-injury, type of contract, previous deployments, gender, age or cohort was found. There were no problems with item fit.

**Table 2.** Global test of fit and differential item function for the 10-item and the 8-item PRIM-Depression scale. Item difficulties for the graphical log-linear Rasch model for the 10-item PRIM-Depression scale.

	10-item PRIM-Depression, RM			10-item PRIM-Depression, GLLRM**			8-item PRIM-Depression, RM		
Tests of fit	CLR	*df*	*p*	CLR	*df*	*p*	CLR	*df*	*p*
Global homogeneity	18.1	9	.03*	18.7	11	.07	14.3	7	.05
Global DIF relative to									
Danger	8.0	9	.53	7.0	11	.80	4.4	7	.73
Contract	7.2	9	.61	15.7	11	.15	6.5	7	.49
Previous deployment	11.5	9	.25	13.2	11	.28	11.4	7	.12
Gender	9.1	9	.43	10.5	11	.49	6.4	7	.49
Age group	16.9	9	.05	22.9	11	.02*	10.7	7	.15
Cohort	29.6	9	< .001	5.7	7	.57	6.0	7	.54

CLR = Conditional likelihood ratio test. *df* = Degrees of freedom. DIF = Differential item function. GLLRM = Graphical log-linear Rasch model. RM = Rasch model.

All *p*-values were adjusted for false discovery rate (FDR) by using the Benjamini-Hochberg procedure.

*p-values that were above the 5% critical limit after the adjustment for FDR

(** The GLLRM for the 10-item PRIM-Depression scale included DIF for item 3 and item 8 relative to cohort; see also Table 3).

**Table 3.** Item difficulties for the graphical log-linear Rasch model for the 10-item PRIM-Depression scale.

Item	Item difficulties in logits, 10-item PRIM-Depression scale	Item difficulties in logits, 8-item PRIM-Depression scale
1	−0.54	−1.15
2	3.76	3.16
3 Cohort 2		
2	−2.06	
3 Cohort 1		
1	−0.94	
4	−2.38	−3.08
5	−0.04	−0.63
6	−0.23	−0.82
7	1.43	0.84
8 Cohort 2		
2	−2.79	
8 Cohort 1		
1	−1.84	
9	1.30	0.72
10	1.55	0.97

The DIF in the GLLRM for the 10-item PRIM-Depression scale indicates that both item 3 (*worries about the future*) and item 8 (*generally worried*) were more difficult to endorse for personnel from Cohort 1 compared to personnel from Cohort 2 (Table 3), i.e. personnel from Cohort 1 needed a higher score on the latent PRIM-Depression scale to endorse these symptoms as being present compared to personnel from Cohort 2. In addition, it is clear that both item 3 and item 8 are rather easy to endorse, with only item 4 (*feeling sad*) being as easy as item 3 and item 8. At the other end of the difficulty spectrum is item 2 (*thoughts about committing suicide*) as by far the most difficult item to endorse independent of the level on the latent PRIM-Depression scale.

We proceeded to evaluate the effect of DIF on the observed scores by testing hypotheses of equality of the observed and equated mean scores of personnel from Cohort 1 to the observed mean scores of personnel from Cohort 2 by using a chi-square test statistic (Table 4). We found that the *observed* mean score of personnel from Cohort 1 was *not* significantly different from the observed mean scores of personnel from Cohort 2. However, the *equated* mean score of Cohort 1 personnel was significantly different from the observed mean scores of Cohort 2 personnel (*p* < .001). As such, the effect of the DIF was great enough to cause a type II error, and we therefore equated the scores for Cohort 1 personnel as presented in Table 5, so that bias-free comparisons could be performed.

**Table 4.** Effect of differential item function on the total score of the 10-item PRIM-Depression scale.

	Observed and DIF-equated total sum scores (#)		
	# Obs Cohort 2	# Obs Cohort 1	# Equated Cohort 1
Mean	3.07	3.51	3.74*
*SD*	2.82	2.86	2.82
SE	0.17	0.16	0.15

DIF = Differential item function. # Obs = observed total score. # Equated = equated total score.

The effect of the cohort-DIF can be seen as the difference between the observed and equated mean scores for Cohort 1 compared to Cohort 2.

* = Hypothesis of equality is rejected for equated mean score Cohort 1 and observed mean score Cohort 2 (*p* < .001).

**Table 5.** Score equation for Cohort 1 to adjust for cohort-differential item function in the 10-item PRIM-Depression scale.

Cohort 2 sum scores	Cohort 1 DIF-equated scores
1.00	1.39
2.00	2.49
3.00	3.44
4.00	4.33
5.00	5.23
6.00	6.14
7.00	7.08
8.00	8.04
9.00	9.01

DIF = Differential item function.

Maximum and minimum scores are not shown, as no equation is possible of extreme scores.

Targeting was rather varied for groups of individuals defined by the degree of danger they have been exposed to, age group and cohort (Table 6). The best targeting of both theta and the observed score, was found for personnel with a high level of exposure to danger, who are below the age of 30 years, and who were deployed with Cohort 2. Poorest targeting was found for personnel with a low level of exposure to danger, who are 30 years or older, and who were deployed with Cohort 1. Accordingly, the full 10-item PRIM-Depression scale is best targeted to young individuals exposed to the toughest conditions during deployment. The reliability of the full 10-item PRIM-Depression scale was very good for all groups of personnel (see Table 6).

**Table 6.** Targeting and reliability of the 10-item and the 8-item PRIM-Depression scale.

	Theta				Sum score			
Groups defined by danger, age group, cohort	Target	Mean	Test inf. Target index	RMSE target index	Target	Mean	Mean SEM	Reliability
	10-item PRIM-Depression scale							
Low, < 30 years, Cohort 2	0.12	−1.94	0.626	0.759	5.23	3.07	0.90	0.89
High, < 30 years, Cohort 2	0.12	−0.41	0.803	0.911	5.23	4.54	1.07	0.84
Low, ≥ 30 years, Cohort 2	0.12	−2.54	0.584	0.724	5.23	2.53	0.86	0.88
High, ≥ 30 years, Cohort 2	0.12	−1.16	0.732	0.870	5.23	3.78	1.00	0.86
Low, < 30 years, Cohort 1	−0.10	−1.58	0.663	0.796	4.63	2.96	0.99	0.84
High, < 30 years, Cohort 1	−0.10	−0.52	0.739	0.810	4.63	4.14	1.07	0.85
Low, ≥ 30 years, Cohort 1	−0.10	−2.61	0.479	0.598	4.63	2.48	0.78	0.91
High, ≥ 30 years, Cohort 1	−0.10	−1.38	0.635	0.769	4.63	3.34	0.96	0.88
Groups defined by danger and gender (*N*)	8-item PRIM-Depression scale							
Low, male (300)	0.00	−2.89	0.477	0.650	4.01	1.73	0.72	0.86
High, male (205)	0.00	−1.45	0.648	0.827	4.01	2.69	0.89	0.83
Low, female (28)	0.00	−1.58	0.598	0.773	4.01	2.64	0.85	0.87

RMSE = The root mean squared error. SEM = The standard error of measurement of the observed score.

Targeting and reliability has been estimated for all groups defined by exogenous variables involved in differential item function (Cohort in the case of the 10-item PRIM-Depression scale) as well as exogenous variables associated with the score (Danger and Age group in the case of the 10-item PRIM-Depression scale, and Danger and Gender in the case of the 8-item PRIM-depression scale).

### 4.2. Item analysis of the reduced 8-item depression scale

Since the full 10-item PRIM-Depression scale did not fit the RM, and evidence of DIF was found relative to item 3 and item 8, we proceeded to analyse an 8-item version of the scale excluding these two items, thereby attempting to eliminate the DIF.

The raw distribution of depression scores ranged from 0 to 8 with a mean of 2.18 (*SD* = 2.24) symptoms being endorsed (data not shown). As for the full 10-item PRIM-Depression scale, no problems were found with monotonicity or homogeneity of the items, and internal consistency was excellent. The reduced 8-item depression scale fit a pure RM, the global test of DIF showed no evidence of DIF (Table 2), and there were no problems with item fit. Having eliminated the two DIF-items (item 3 and item 8), the item difficulties of the remaining eight items shifted somewhat (Table 3). However, item 4 (*feeling sad*) remained the easiest item to endorse and item 2 (*thoughts about committing suicide*) the most difficult.

Targeting was rather varied for the different groups of personnel defined by the exogenous variables associated with the depression score, i.e. high/low danger exposure and gender (Table 6). When broken down into all these subgroups, the best targeting, of both theta and the observed score, was found for males with high level of exposure to danger. The poorest targeting was found for males with low level of exposure to danger, while females with low level of exposure to danger where almost as well-targeted as males with a high level of danger exposure. Accordingly, the reduced 8-item depression scale is best at targeting males exposed to relatively high levels of danger and females exposed to relatively low levels of danger. The reliability of the depression scale was very good for all subgroups.

Since the 8-item version of PRIM-Depression fit a RM, we proceeded to estimate the correlation of this scale with BDI (for Cohort 1) and DASS-Depression (for Cohort 2). We found a correlation of 0.68 (*p *< .001) with the BDI for Cohort 1, and a correlation of 0.74 (*p *< .001) with DASS for Cohort 2.

### 4.3. Analysis of cutoff score for screening

The analysis using a cutoff score of 5 showed that item parameters were *not* equal across score groups (CLR 28.4, *df* 11, *p* < .01) in the GLLRM including DIF for item 3 and item 8 relative to cohort. This indicates a qualitative difference in depression between those with a score from 0–5 and those with a score from 6–10. To explore this difference further, we proceeded to analyse each of the score groups separately using Rasch analysis and graphical log-linear Rasch analysis.

The analysis of the 0–5 score group showed that item responses among the low-scoring individuals fit a GLLRM with DIF for item 3 and item 8 relative to Cohort and no further DIF or LD, as was the case in the analysis of the entire score range (CLR 8.6, *df* 11, *p* = .66). There were no problems with item fits.

The analysis of the 6–10 score group showed that item responses for high-scoring individuals fit a RM (CLR 10.1, *df* 8, *p* = .26), except for a slight problem with the fit of item 4. The problem with item 4 was that there was no variance on this item since all individuals endorsed this item. Hence, we cannot properly assess fit of this item to the RM.

That items of the low-scoring individuals have DIF for two items and fit a GLLRM while items for the high-scoring individuals have no DIF and fit a RM, suggests that score equation is only necessary for low-scoring individuals, while the raw scale score is actually sufficient for the high-scoring individuals. Further analyses of the low-scoring individuals confirmed that this was the case.

## 5. Discussion

In this study, we have investigated the validity of the 10-item PRIM-Depression scale developed for the assessment of depression in Danish military personnel following deployment, using Rasch measurement models.

In the initial analyses of all 10 PRIM-Depression items, we found an excellent overall consistency and no problems with monotonicity or homogeneity. However, the 10-item PRIM-Depression did not fit the pure RM. When testing the fit of items to a GLLRM, we found evidence of DIF for item 3 and item 8 relative to cohort. However, fit to a GLLRM with the two mentioned instances of DIF was established. Therefore, we proceeded to analyse an 8-item version, excluding the two items causing DIF in the full scale. In brief, the 8-item version fitted a pure RM, and we found no problems with DIF relative to any of the exogenous variables. The 8-item version correlated strongly with other depression measures.

In the literature, rates of depression among soldiers following deployment to war zones have been found to differ across studies (Hoge et al., 2004; Reijnen et al., 2015). While differences in rates of depression might reflect actual differences in mental health toll across different nations, wars and missions, it might also partly reflect measurement difficulties, such as DIF relative to cohort, that are not accounted for. To the best of our knowledge, no prior studies have tested the validity of depression measures in military samples using RM or other instantiations of IRT. Looking at other trauma samples, a few studies have investigated the cross-cultural validity of a depression measure (Choi, Mericle, & Harachi, 2006) and item overlap between depression and PTSD (Elhai et al., 2011) using RM. Since these studies are not specifically testing for DIF, previous literature cannot confirm whether cohort DIF poses a challenge in military studies investigating depression. Based on our results, cohort DIF is suggested to be a measurement issue that needs to be addressed. Indeed, we found that that the effect of DIF in the 10-item version was great enough to cause a type II error, in that the raw depression scores for the two cohorts were not significantly different, although they were indeed different when comparing the equated score of Cohort 1 with the raw score of Cohort 2. Hence, this is a very good example of the importance of score equation when DIF is present, in that actual differences in depression between cohorts would otherwise have gone unnoticed.

With regard to targeting, we found that the 10-item PRIM-Depression scale was best targeted for military personnel who were young (< 30 years), who were exposed to high levels of danger during their mission, and who belonged to Cohort 2. Age has previously been identified as a risk factor for PTSD (Brewin et al., 2000), but a recent meta-analysis found age at time of trauma to be largely unrelated to military PTSD (Xue et al., 2015). While there remains no conclusive evidence concerning age, exposure to danger is presumably stressful. Hence, when targeting cannot be equal for all, it is desirable that it should be optimal for those who might be at greater risk for developing depressive symptoms following deployment, i.e. those who are younger and exposed to more danger. Our findings show that PRIM-Depression is most precise for individuals who might indeed have an increased risk of developing depression following deployment. This suggests that the 10-item PRIM-Depression scale is suitable for screening military personnel after return from deployment.

The fact that the 8-item version fit a pure RM suggest that this reduced version of the scale obtains a raw measure of depression that can be used across cohorts without score equation. Hence, the 8-item version is an adept screening tool that can readily be applied to military personnel returning from deployment regardless of the cohort they belonged to or the mission to which they were deployed.

With regards to targeting, the 8-item depression scale was best targeted for males with high exposure to danger. As for the 10-item version, this suggests that the depression scale is most precise for those at greatest risk for developing depression symptoms due to exposure to danger. Curiously however, this was not the case for females, where the 8-item depression scale was best targeted for those with low exposure to danger. This finding is in line with a recent study of American Iraq and Afghanistan soldiers, which found that females are generally at greater risk for developing symptoms of depression following deployment than males (Haskell et al., 2010). Hence, a tentative explanation of this sex-difference may be that lower levels of exposure are needed to be at risk for depression symptoms for females, which makes targeting of the depression scale good for females even at low levels of exposure. This remains speculative, however, and more research is needed to confirm this explanation.

For screening purposes, a cutoff score to be used for identification of probable cases is of great importance. Hence, we tested whether a cutoff score of 5 on the 10-item version could be used to divide the cohort into groups of low (5 and below) or high (6 and above) depression-symptomatology. We approached this by establishing a fit to different models for low- and high-scorers, respectively. For the low-scoring group, the scale did not fit a RM but, instead, a GLLRM with DIF for item 3 and item 8, exactly like for the entire sample. Conversely, when we tested the high-scoring group, items fitted the RM. Based on the cutoff score analysis in this sample, however, we cautiously suggest that on the version of the PRIM-Depression scale tested here (i.e. the 10-item version with dichotomous items), having a score of 6 or higher indicates a need for further assessment of depression symptoms by a psychologist. We did not test a cutoff score for the 8-item version with dichotomous items, since we could not directly translate a cutoff score from the original 10-item ordinal scare to the 8-item dichotomous scale. However, in future endeavours, such a cutoff score should be identified and tested.

The present study has certain limitations. First, we included only two cohorts, for which comparisons were made. Ideally, we would compare the depression across multiple cohorts, but we have extensive data on the relevant covariates for these two cohorts only and, therefore, we included only these cohorts. However, according to recent research, the sample had sufficient statistical power for estimation of RM (Draxler & Alexandrowicz, 2015). Regarding the depression items themselves, we do not have exact knowledge on the source of each individual item and how they were selected. However, comparison of the items with, for instance, the depression items included in DASS21 (Lovibond & Lovibond, 1995) indicates semantic overlap. Finally, additional exogenous variables, such as more detailed information on deployment characteristics, could have been relevant to include in the RM, but they were not available.

A major strength of the study is the use of RM (Rasch, 1960), which enables us to test ideal measurement of PRIM-Depression; i.e. whether the score is a sufficient statistic containing all information in responses on the depression scale (Kreiner, 2013, 2007). With very few exceptions (Choi et al., 2006; Elhai et al., 2011), RM has not been used to validate depression measures in military trauma samples.

In conclusion, we find that the PRIM-Depression scale constitutes a relevant measure of depression in Danish military personnel deployed to war zones with the Danish Military. Whereas the 10-item PRIM-Depression scale can be used with appropriate score equation, the reduced 8-item version provides a reliable measure for comparison without score equation. When investigating levels of depression in one cohort, the 10-item version might be preferred, since more items generally increase accuracy of a measure. However, when comparing levels of depression across cohorts, our results imply that the 8-item version should be preferred since the raw score of the scale is comparable between cohorts without score equation. Comparability across cohorts is of great importance for screening purposes as well as for research on the psychological toll of different missions and wars and, as such, the 8-item version is a valuable tool that can easily be implemented.

We recommend that future research include more cohorts to ensure that potential DIF is detected and differences in levels of depression across cohorts represent actual and not methodological differences.

### 5.1. Implications

With the use of the validated 8-item PRIM-Depression scale, retrospective data of all deployed Danish soldiers from 1998 onwards can be analysed to gain insights of deployment-related depression. Soldiers coming home from future deployments can be screened using the 8-item PRIM-Depression scale, ensuring identification and follow-up of soldiers with possible deployment-related depression.

## Highlights

- A measure of depression-symptoms (PRIM-Depression) has been distributed six months after home-coming to all Danish soldiers who have deployed to international missions since 1998
- Here, the PRIM-Depression is validated using Rasch measurements models with the specific aim of testing differential item functioning across deployment cohorts.
- We find that the full 10-item version of PRIM-Depression is reliable only with score equation, whereas a reduced 8-item version is valid across cohorts without score equation
- Hence, the 8-item PRIM-Depression scale provides a valid tool for comparing levels of depression for deployments that differ in level of threat and combat exposure.
